# IScore, a useful prognostic tool for patients with acute ischemic stroke treated with intravenous thrombolysis: a validation study

**DOI:** 10.1055/s-0042-1758397

**Published:** 2023-03-22

**Authors:** Valéria Cristina Scavasine, Rebeca Teixeira Costa, Viviane de Hiroki Flumignan Zétola, Marcos Christiano Lange

**Affiliations:** 1 Universidade Federal do Paraná, Hospital de Clínicas, Divisão de Neurologia, Curitiba PR, Brasil.

**Keywords:** Cerebrovascular Disorders, Ischemic Stroke, Thrombolytic Therapy, Validation Study, Prognosis, Transtornos Cerebrovasculares, AVC Isquêmico, Terapia Trombolítica, Estudo de Validação, Prognóstico

## Abstract

**Background** Stroke is one of the major causes of disability and mortality worldwide. Up to 30% of individuals who experience stroke die within 30 days, and more than 50% of those who survive will have some degree of disability. There are some predetermining factors based on admission data that could be used to objectively assess the odds of poor outcomes, including the Ischemic Stroke Predictive Risk Score (IScore).

**Objective** To analyze and validate the IScore in patients undergoing intravenous thrombolysis for stroke and compare the results of this predictor with actual death and disability outcomes.

**Methods** In a retrospective study, data were collected from a database housed at the Stroke Unit of the Teaching Hospital of Universidade Federal do Paraná, Southern Brazil. The IScore was applied to admission data from 239 patients, and the results were compared with actual outcomes (death and disability) within 30 days and 1 year after the stroke event. Data analysis was performed using an analysis of the receiver operating characteristic (ROC) curve to determine the sensitivity and specificity of the IScore in the study population.

**Results** The IScore demonstrated moderate sensitivity and high specificity in patients with stroke who underwent thrombolysis when evaluated after 30 days and 1 year of the event.

**Conclusions** The IScore can be applied to in stroke patients undergoing thrombolysis; therefore, it may be used as an objective prognostic tool to guide clinical decision-making. Understanding the prognosis of patients in the acute phase can assist clinicians in making the best therapeutic decisions and enable better end-of-life care.

## Resumo

**Antecedentes** O acidente vascular cerebral (AVC) é uma das principais causas de morte e incapacidade no mundo. Até 30% dos indivíduos evoluem a óbito nos primeiros 30 dias. Dos sobreviventes, mais da metade ficam com alguma sequela. Certas variáveis identificadas já na admissão podem sinalizar maior risco de desfecho desfavorável. Uma dessas ferramentas prognósticas é a Escala de Previsão de Riscos de AVC Isquêmico (Ischemic Stroke Predictive Risk Score, IScore, em inglês).

**Objetivo** Analisar e validar a aplicação do IScore em pacientes com AVC isquêmico submetidos a trombólise endovenosa e comparar os resultados obtidos com a aplicação da escala aos verdadeiros desfechos de morte e incapacidade funcional.

**Métodos** Trata-se de um estudo retrospectivo, cujos dados foram obtidos do banco de dados da Unidade de AVC do Hospital de Clínicas da Universidade Federal do Paraná. A IScore foi aplicada conforme os dados de admissão de 239 pacientes. Os pacientes foram acompanhados ambulatorialmente, e os resultados da escala foram comparados aos desfechos reais de mortalidade e incapacidade. Os resultados foram demonstrados por meio da curva característica de operação do receptor ( *receiver operating characteristic* , ROC, em inglês) para determinar sensibilidade e especificidade da escala.

**Resultados** Em pacientes com AVC submetidos a trombólise, a IScore demonstrou moderada sensibilidade e alta especificidade para predizer desfechos tanto de óbito quanto de incapacidade, nos intervalos de 30 dias e 1 ano após o evento.

**Conclusões** A IScore pode ser aplicada em pacientes com AVC isquêmico, na população específica submetida a trombólise; portanto, pode ser usada como uma ferramenta prognóstica útil, que pode guiar decisões terapêuticas. Entender o prognóstico de paciente na fase aguda pode auxiliar os clínicos a fazer as melhores decisões terapêuticas e possibilitar melhores cuidados ao fim da vida.

## INTRODUCTION

Stroke is a major cause of disability worldwide and the second leading cause of death in Brazil. 1 Despite recent advances in the treatment of the acute phase of stroke, death and disability remain common outcomes, with more than 50% of patients surviving with some degree of dependence. 2

The Ischemic Stroke Predictive Risk Score (IScore) is a predictor of the risk of death and disability at 30 days and 1 year after an acute stroke. It was validated in 2011 3 based on data from patients who experienced ischemic stroke, regardless of the type of treatment administered in the acute phase. The factors related to the worst outcome of this study 3 included age, sex, stroke severity, smoking, atrial fibrillation, congestive heart failure, cancer, kidney disease on dialysis, and hyperglycemia at admission.

It is not easy to predict clinical outcomes in stroke patients who undergo thrombolysis, even for neurologists with expertise in stroke treatment. In the Clinician JUdgment vs Risk Score to predict Stroke outComes (JURaSSiC) study, 4 only one in six clinicians demonstrated accuracy in predicting death or disability at discharge. In this scenario, an appropriate scoring system may help physicians in making acute treatment decisions.

In the present study, we aimed to replicate these results in a real-world Brazilian public referral service and in patients undergoing intravenous thrombolysis for stroke treatment, to be able to objectively assess the prognosis of patients as early as possible.

## METHODS

The present is a retrospective cohort study. Data were collected from the database of the Stroke Unit of the Teaching Hospital of Universidade Federal do Paraná, Southern Brazil. We included 239 patients who underwent thrombolytic therapy admitted to the Stroke Unit between March 1, 2010, and December 31, 2015. The study was approved by the local Ethics Committee, and the board waived the need for patient consent.

**Figure 1:**
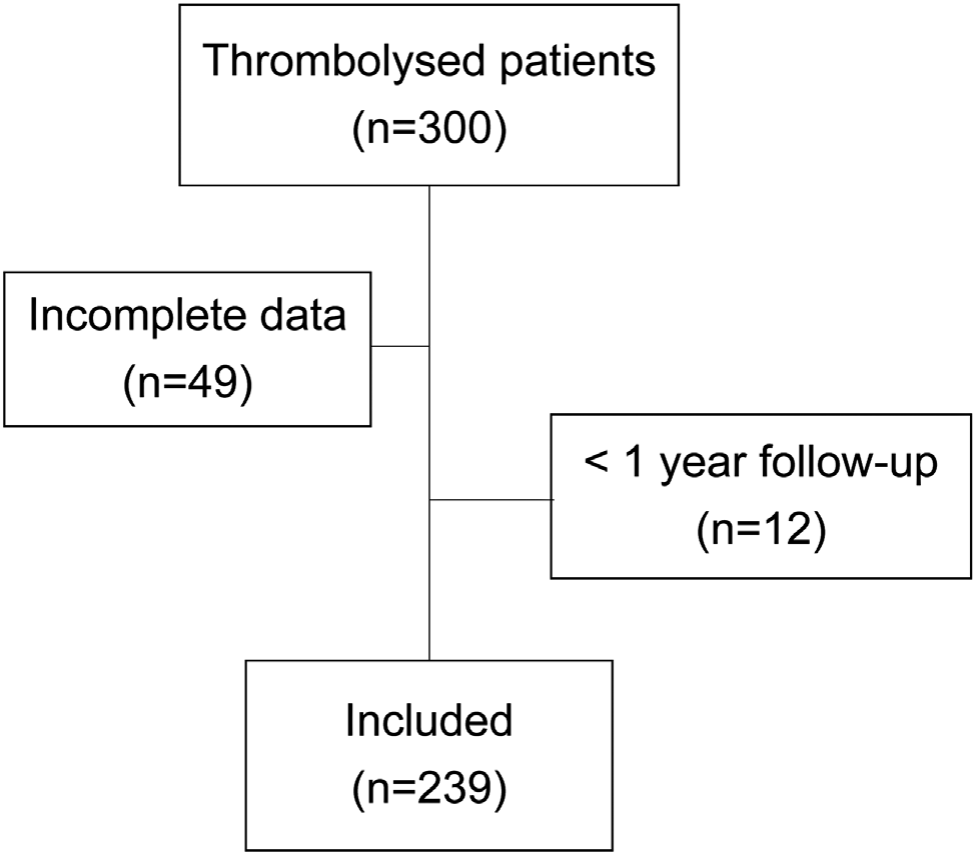
Flowchart of the data collection process.

The inclusion criteria were as follows: admission within 24 hours of symptom onset; ischemic stroke treated with intravenous thrombolysis; available information regarding the National Institutes of Health Stroke Scale/Score (NIHSS) at admission and etiological classification of stroke, and all the data necessary to apply the risk score. Stroke-mechanism subtypes were defined according to the Stop-Stroke Study-Trial of Org 10172 in Acute Stroke Treatment (SSS-TOAST) classification system as follows: large-artery atherosclerosis; cardioembolism; small-vessel occlusion; stroke of other determined etiology; and stroke of undetermined etiology. 5 Individuals with less than 1 year of follow-up after the event, those not eligible for intravenous thrombolysis, and those with lack of admission data necessary to apply the IScore were excluded. Of the 300 patients submitted to thrombolysis, 49 were excluded due to lack of data necessary to apply the IScore, and 12 were excluded due to loss of follow-up in less than 1 year after the stroke ( Figure 1 ).

Data were collected retrospectively from a review of an existing database in the service. Those responsible for the data collection were the second-year neurology residents who admitted and followed up these patients during hospitalization in a stroke unit and later in outpatient follow-up consultations. The patients underwent follow-up in regular routine consultations at the vascular neurology outpatient clinic of the service.

The etiological investigation of stroke was performed in all patients with the following tests: transthoracic echocardiography, cranial and cervical arterial computed tomography (CT) angiography, electrocardiogram, 24-hour Holter, and, when necessary, cranial resonance, transcranial Doppler, and transesophageal echocardiography. The patients were then discharged with a complete investigation and etiological classification, except those with a negative investigation, who were then classified as having indeterminate or cryptogenic stroke.

For the IScore, we used admission data to predict the risk of morbidity and mortality, so the diagnostic data on diabetes, smoking, alcohol consumption, congestive heart failure, cancer, chronic kidney disease, for example, had to be obtained prior to the event, collected in the anamnesis with a family member or patient.

The IScore protocol was applied according to admission data. The results were compared with the actual cause of death and disability at 30 days and 1 year after the stroke event.

Receiver operating characteristic (ROC) curve analysis was used to assess the accuracy of the IScore in the study population. The ROC curve illustrates the exchange between the true-positive fraction and false-positive fraction as an alteration of the positivity criterion, and makes it possible to establish the sensitivity and specificity of the method in the study population.

The degree of disability was assessed using the modified Rankin Scale (mRS), with scores from 0 to 2 considered an independent or favorable outcome, and from 3 to 6 defined as an unfavorable outcome. 6

## RESULTS

A total of 239 patients were included in the present study, among whom 122 (51%) were female and 117 (49%) were male. The most prevalent risk factor was arterial hypertension, which was present in 70.7% of the sample. Almost all patients (98%) were previously independent, with an mRS score between 0 and 1. The baseline characteristics are summarized in Table 1 . The leading poststroke complication was intracranial hemorrhage, which occurred in ∼ 20% of patients, followed by pneumonia in 13%.

**Table 1:** Baseline characteristics

Variable	N = 239 n (%) or mean
Female, n (%)	122 (51)
Age, mean	62
Previous mRS 0–1, n (%)	120 (98.3)
Hypertension, n (%)	169 (70.7)
Diabetes, n (%)	57 (23.8)
CHD, n (%)	21 (8.8)
Kidney disease on dialysis, n (%)	1 (0.41)
Admission glycemia, mean	140mg/dL
Admission NIHSS, mean	12
Hyperlipidemia, n (%)	63 (26.4)
Alcohol consumption, n (%)	25 (10.5)
Current smoking, n (%)	63 (26.4)
Previous stroke, n (%)	39 (17.3)
CHF, n (%)	26 (10.9)
AF, n (%)	17 (7.1)
Stroke subtype, n (%)	Large artery – 61 (25.5)
	Small vessel – 16 (6.6)
	Cardioembolic – 75 (31.3)
	Other etiologies – 3 (1.2)
	Dissection - 2 (0.83)
	Undetermined – 82 (34.3)

Abbreviations: AF, atrial fibrillation; CHD, coronary heart disease; CHF, congestive heart failure; mRS, modified Rankin Scale; NIHSS, National Institutes of Health Stroke Scale/Score.

**Figure 2:**
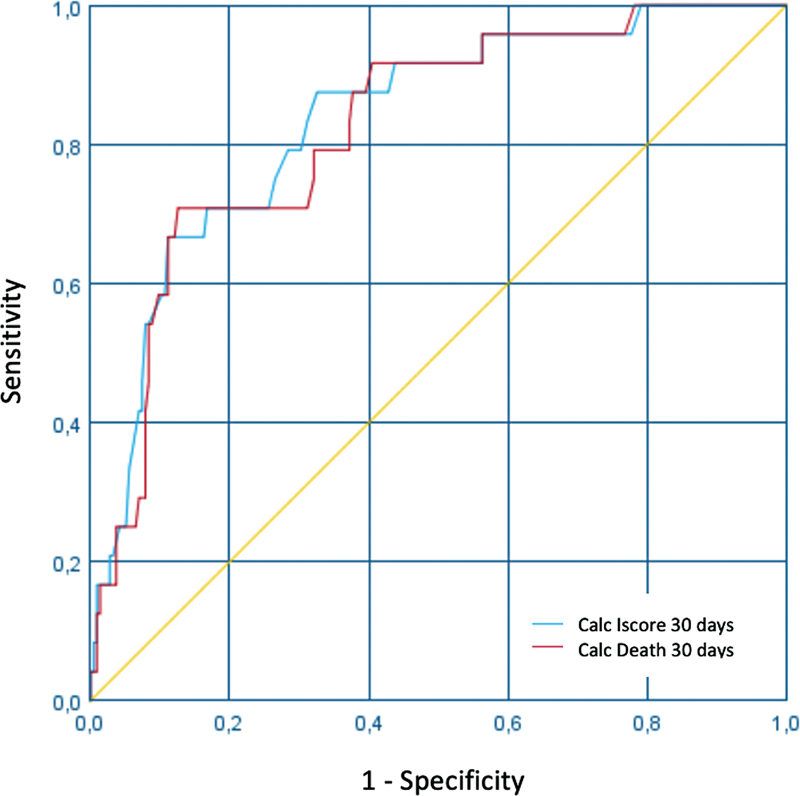
ROC curve demonstrating the correlation between the punctuation on the IScore and death in 30 days.

**Figure 3:**
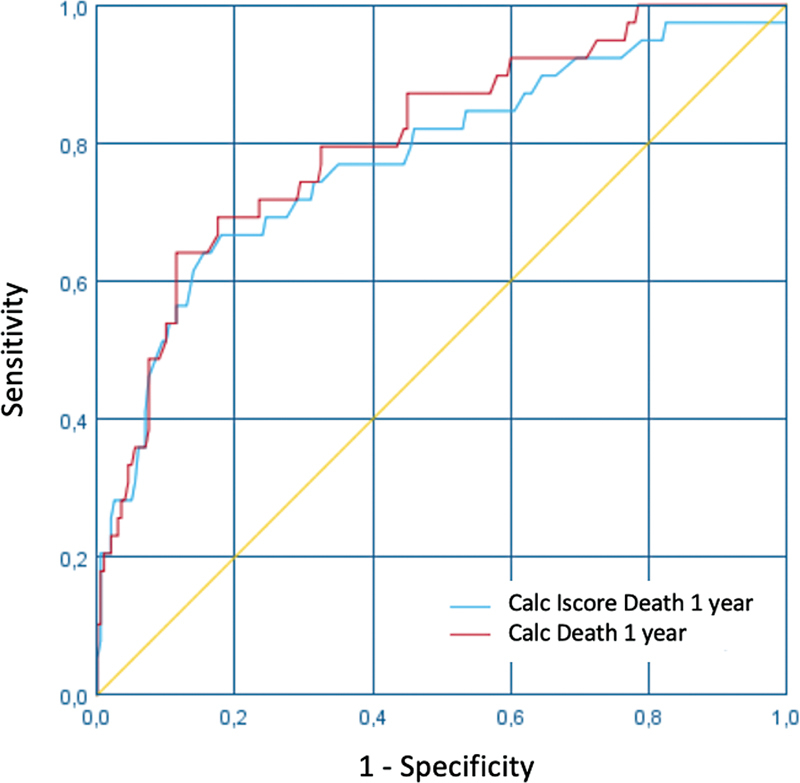
ROC curve demonstrating the correlation between the punctuation on the IScore and death in 1 year.

The IScore demonstrated a sensitivity of 66.7% and specificity of 88.8% for death within 30 days. For disability eithin 30 days, the sensitivity was of 64%, with a specificity of 78.9% The area under the ROC curve (AUC) demonstrated good accuracy for the method, generating a value greater than 0.75 in all studied outcomes ( Figures 2 and 3 ).

In the assessment of death and disability 1 year after the stroke event, the sensitivity was of 64%, while the specificity was of 88.5% ( Table 2 ).

**Table 2:** Sensitivity and specificity of the Ischemic Stroke Predictive Risk Score (IScore) in patients with ischemic stroke submitted to intravenous thrombolysis

	Sensitivity	Specificity
Death within 30 days	66.7%	88.8%
Death/Disability within 30 days	64%	78.9%
Death within 1 year	64%	88.5%

## DISCUSSION

In the present study, the IScore demonstrated high specificity and moderate sensitivity compared with the actual outcomes at 30 days and 1 year after stroke in patients who underwent intravenous thrombolysis.

Almost all patients included in the present study were previously independent, although 10% died within 30 days. In the original publication of the IScore, the mortality rates were of 12% at 30 days and of 22% at 1 year. It has been estimated that up to 30% of individuals who experience stroke die within 30 days of the event. 7 8 In our sample, the mortality rates were lower, possibly due to the selection of patients eligible for thrombolytic therapy.

Previous scores have been developed to help physicians make acute treatment decisions, with some for general acute stroke populations ([ASTRAL], 9 [VISTA] 10 ) and others specific to patients submitted to thrombolysis (DRAGON, 11 [SEDAN], 12 [SPAN-100] 13 ); however, these scores are rarely used in the clinical practice.

The possibility of determining the prognosis at an early stage can facilitate the clinical decision-making and improve the long-term quality of life. Although a cut-off has not yet been established, it has been demonstrated that patients with an IScore ≥ 200 may not benefit from thrombolytic therapy. 14

The limitations of the present study include its retrospective, single-center design, restricted sample of patients with cancer and dialysis, and no clear cut-off point defined by the IScore. However, to our knowledge, this is the first investigation to evaluate the application of the IScore in the specific population of patients undergoing thrombolytic therapy.

This prognostic score may help clinicians make decisions, especially regarding end-of-life care. For patients who experience severe ischemic stroke, supportive care is as necessary as treatment in the acute phase. The prediction of morbidity and mortality can help coordinate the most appropriate rehabilitation strategies and early discussions with the patient and family about frailty and the final stages of life. Anticipating unfavorable outcomes with higher precision may benefit patients and their families by reducing ineffective treatments and promoting high-quality palliative and end-of-life cares.
